# (6*S*,7*S*,8*S*,8a*S*)-6-Ethyl-7,8-dihy­droxy-1,5,6,7,8,8a-hexa­hydro­indolizin-3(2*H*)-one monohydrate

**DOI:** 10.1107/S1600536810044855

**Published:** 2010-11-10

**Authors:** Viktor Vrábel, Ľubomír Švorc, Peter Šafář, Jozefína Žúžiová

**Affiliations:** a Institute of Analytical Chemistry, Faculty of Chemical and Food Technology, Slovak Technical University, Radlinského 9, SK-812 37 Bratislava, Slovak Republic; b Institute of Organic Chemistry, Catalysis and Petrochemistry, Faculty of Chemical and Food Technology, Slovak Technical University, Radlinského 9, SK-812 37 Bratislava, Slovak Republic

## Abstract

The absolute configuration of the title compound, C_10_H_17_NO_3_·H_2_O, was assigned from the synthesis. In the mol­ecular structure, the central six-membered ring of the indolizine moiety adopts a chair conformation, with two atoms displaced by −0.578 (2) and 0.651 (1) Å from the plane of the other four atoms [maximum deviation 0.019 (2) Å] The conformation of the fused oxopyrrolidine ring is close to that of a flat envelope, with the flap atom displaced by 0.294 (1) Å from the plane through the remaining four atoms. In the crystal, one of the hy­droxy groups is hydrogen-bonded to two water mol­ecules, while the other hy­droxy group exhibits an inter­molecular hydrogen bond to the carbonyl O atom, resulting in a chain parallel to the *b* axis.

## Related literature

For the uses of indolizine-based mol­ecules, see: Weidner *et al.* (1989 ▶); Jaung & Jung (2003 ▶); Rotaru *et al.* (2005 ▶); Saeva & Luss (1988 ▶); Kelin *et al.* (2001 ▶). For biological activities of indolizines, see: Oslund *et al.* (2008 ▶); Asano *et al.* (2000 ▶); Tielmann & Hoenke (2006 ▶). For synthesis, see: Šafař *et al.* (2010 ▶). For ring-puckering and conformational analysis, see: Cremer & Pople (1975 ▶); Nardelli (1983 ▶). 
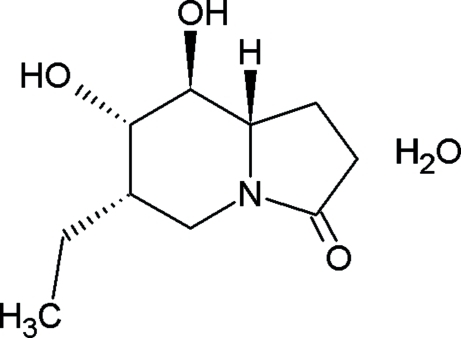


## Experimental

### 

#### Crystal data

C_10_H_17_NO_3_·H_2_O
*M* *_r_* = 217.26
Orthorhombic, 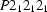

*a* = 7.1398 (3) Å
*b* = 7.3169 (2) Å
*c* = 20.8466 (9) Å
*V* = 1089.05 (7) Å^3^
*Z* = 4
Mo *K*α radiation
μ = 0.10 mm^−1^
*T* = 298 K
0.55 × 0.25 × 0.09 mm

#### Data collection

Oxford Gemini R CCD diffractometer
Absorption correction: analytical (Clark & Reid, 1995 ▶) *T*_min_ = 0.945, *T*_max_ = 0.991
17428 measured reflections
1308 independent reflections
1161 reflections with *I* > 2σ(*I*)
*R*_int_ = 0.028

#### Refinement

*R*[*F* ^2^ > 2σ(*F* ^2^)] = 0.029
*wR*(*F* ^2^) = 0.081
*S* = 1.06
1308 reflections
148 parameters
H atoms treated by a mixture of independent and constrained refinement
Δρ_max_ = 0.16 e Å^−3^
Δρ_min_ = −0.14 e Å^−3^

### 

Data collection: *CrysAlis CCD* (Oxford Diffraction, 2006 ▶); cell refinement: *CrysAlis RED* (Oxford Diffraction, 2006 ▶); data reduction: *CrysAlis RED*; program(s) used to solve structure: *SHELXS97* (Sheldrick, 2008 ▶); program(s) used to refine structure: *SHELXL97* (Sheldrick, 2008 ▶); molecular graphics: *DIAMOND* (Brandenburg, 2001 ▶); software used to prepare material for publication: *enCIFer* (Allen *et al.*, 2004 ▶) and *PLATON* (Spek, 2009 ▶).

## Supplementary Material

Crystal structure: contains datablocks I, global. DOI: 10.1107/S1600536810044855/fj2360sup1.cif

Structure factors: contains datablocks I. DOI: 10.1107/S1600536810044855/fj2360Isup2.hkl

Additional supplementary materials:  crystallographic information; 3D view; checkCIF report

## Figures and Tables

**Table 1:** Hydrogen-bond geometry (Å, °)

*D*—H⋯*A*	*D*—H	H⋯*A*	*D*⋯*A*	*D*—H⋯*A*
O22—H22*A*⋯O21^i^	0.82 (3)	1.94 (3)	2.7505 (19)	173 (3)
O24—H24*A*⋯O22^ii^	0.91 (3)	2.07 (3)	2.919 (2)	155 (2)
O24—H24*B*⋯O23^iii^	0.78 (3)	2.14 (3)	2.907 (2)	167 (3)

Symmetry codes: (i) 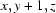; (ii) 
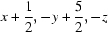
; (iii) 
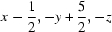
.

**Fig. 1.**
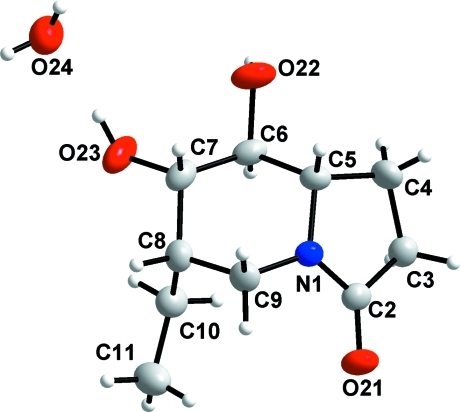
Molecular structure of (I) with the atomic numbering scheme. Displacement ellipsoids are drawn at the 50% probability level (Brandenburg, 2001).

**Fig. 2.**
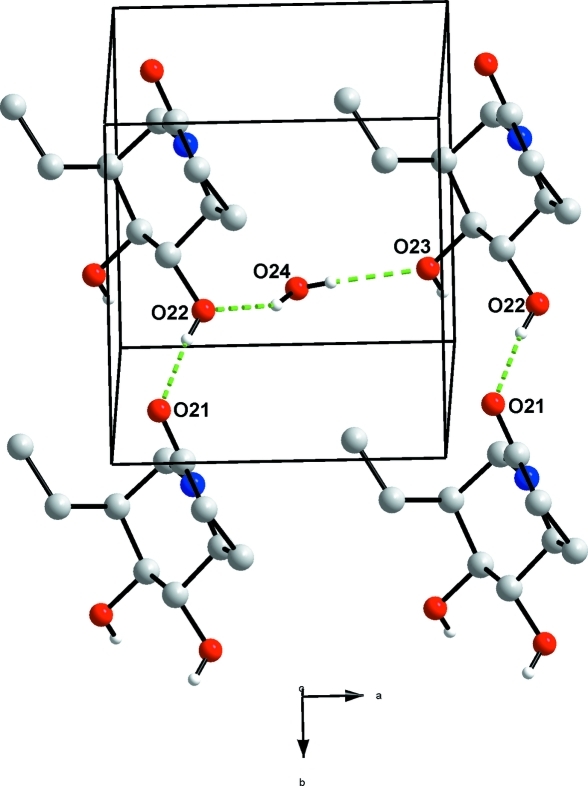
A packing of the molecule of (I), viewed along the b axis.

## supplementary crystallographic information

### Comment

Indolizines based molecules are known for their use as synthetic dyes (Weidner *et al.*, 1989; Jaung & Jung, 2003), fluorescent materials (Rotaru *et al.*, 2005; Saeva & Luss, 1988) and also as key intermediates for the synthesis of indolizine based molecules (Kelin *et al.*, 2001). Indolizines both synthetic and natural have also been ascribed with a number of useful biological activities (Oslund *et al.*, 2008; Asano *et al.*, 2000; Tielmann & Hoenke, 2006) such as antibacterial, antiviral, CNS depressants, anti-HIV, anti-cancer and have been used for treating cardiovascular ailments.

Due to the diverse properties of indolizine derivatives, the structure of the title compound, (I), has been determined as part of our study of the conformational changes caused by different substituents at various positions on the indolizine ring system. The absolute configuration was established by synthesis and is depicted in the scheme and figure. The expected stereochemistry of atoms C5, C6, C7 and C8 was confirmed as *S*, *S*, *S* and *S*, respectively (Fig. 1). The central six-membered ring is not planar and adopts a chair conformation (Cremer & Pople, 1975). A calculation of least-squares planes shows that this ring is puckered in such a manner that the four atoms C5, C6, C8 and C9 are coplanar to within 0.019 (2) Å, while atoms N1 and C7 are displaced from this plane on opposite sides, with out-of-plane displacements of -0.578 (2) and 0.651 (1) Å, respectively. The oxopyrrolidine ring attached to the indolizine ring system has flat-envelope conformation with atom C4 on the flap (Nardelli, 1983). The deviation of atom C4 from the mean plane of the remaining four atoms N1/C2/C3/C5 is 0.294 (1) Å. The N1—C5 and N1—C9 bonds are approximately equivalent and both are much longer than the N1—C2 bond. Atom N1 is *sp*^2^-hybridized, as evidenced by the sum of the valence angles around it (358.4 (2)°). These data are consistent with conjugation of the lone-pair electrons on N1 with the adjacent carbonyl C2═O21. The H atoms (H24A and H24B) of the water molecule form O—H···O intermolecular hydrogen bonds with the O atoms (O22 and O23) of both present hydroxy groups, which may, in part, influence the molecular configuration. There have been observed also another intermolecular O—H···O hydrogen bonds, in which carbonyl oxygen O21 participates as acceptor and atom O22 as donator (Table 1). All the interactions demonstrated were found by *PLATON* (Spek, 2009).

### Experimental

The title compound 6S,7*S*,8S,8aS)-6-ethyl-7,8-dihydroxyhexahydroindolizin-3(2*H*)-one monohydrate was prepared according literature procedures of Šafař *et al.* (2010).

### Refinement

All H atoms were placed in geometrically idealized positions and constrained to ride on their parent atoms, with C—H distances in the range 0.93–0.98 Å and O—H distance 0.85 Å and *U*_iso_ set at 1.2*U*_eq_ of the parent atom. The absolute configuration could not be reliably determined for this compound using Mo radiation, and has been assigned according to the synthesis; 907 total Friedel pairs have been merged. Due to the absence of anomalous dispersion the Flack parameter was not refined

### Crystal data

**Table:**

C_10_H_17_NO_3_·H_2_O	*F*(000) = 472
*M**_r_* = 217.26	*D*_x_ = 1.325 Mg m^−^^3^
Orthorhombic, *P*2_1_2_1_2_1_	Mo *K*α radiation, λ = 0.71073 Å
Hall symbol: P 2ac 2ab	Cell parameters from 10421 reflections
*a* = 7.1398 (3) Å	θ = 3.4–29.5°
*b* = 7.3169 (2) Å	µ = 0.10 mm^−^^1^
*c* = 20.8466 (9) Å	*T* = 298 K
*V* = 1089.05 (7) Å^3^	Prism, colourless
*Z* = 4	0.55 × 0.25 × 0.09 mm

### Data collection

**Table:**

Oxford Gemini R CCD diffractometer	1308 independent reflections
Radiation source: fine-focus sealed tube	1161 reflections with *I* > 2σ(*I*)
graphite	*R*_int_ = 0.028
Detector resolution: 10.4340 pixels mm^-1^	θ_max_ = 26.4°, θ_min_ = 4.1°
Rotation method data acquisition using ω and φ scans	*h* = −8→8
Absorption correction: analytical (Clark & Reid, 1995)	*k* = −9→9
*T*_min_ = 0.945, *T*_max_ = 0.991	*l* = −26→26
17428 measured reflections	

### Refinement

**Table:**

Refinement on *F*^2^	Primary atom site location: structure-invariant direct methods
Least-squares matrix: full	Secondary atom site location: difference Fourier map
*R*[*F*^2^ > 2σ(*F*^2^)] = 0.029	Hydrogen site location: inferred from neighbouring sites
*wR*(*F*^2^) = 0.081	H atoms treated by a mixture of independent and constrained refinement
*S* = 1.06	*w* = 1/[σ^2^(*F*_o_^2^) + (0.0511*P*)^2^ + 0.0923*P*] where *P* = (*F*_o_^2^ + 2*F*_c_^2^)/3
1308 reflections	(Δ/σ)_max_ < 0.001
148 parameters	Δρ_max_ = 0.16 e Å^−^^3^
0 restraints	Δρ_min_ = −0.14 e Å^−^^3^

### Special details

**Table:**

Experimental. face-indexed (Oxford Diffraction, 2006)
Geometry. All e.s.d.'s (except the e.s.d. in the dihedral angle between two l.s. planes) are estimated using the full covariance matrix. The cell e.s.d.'s are taken into account individually in the estimation of e.s.d.'s in distances, angles and torsion angles; correlations between e.s.d.'s in cell parameters are only used when they are defined by crystal symmetry. An approximate (isotropic) treatment of cell e.s.d.'s is used for estimating e.s.d.'s involving l.s. planes.
Refinement. Refinement of *F*^2^ against ALL reflections. The weighted *R*-factor *wR* and goodness of fit *S* are based on *F*^2^, conventional *R*-factors *R* are based on *F*, with *F* set to zero for negative *F*^2^. The threshold expression of *F*^2^ > σ(*F*^2^) is used only for calculating *R*-factors(gt) *etc*. and is not relevant to the choice of reflections for refinement. *R*-factors based on *F*^2^ are statistically about twice as large as those based on *F*, and *R*- factors based on ALL data will be even larger.

### Fractional atomic coordinates and isotropic or equivalent isotropic displacement parameters (Å^2^)

**Table:**

	*x*	*y*	*z*	*U*_iso_*/*U*_eq_	
C2	0.7971 (2)	0.5950 (2)	0.21098 (8)	0.0301 (4)	
C3	0.7338 (3)	0.7182 (2)	0.26470 (8)	0.0347 (4)	
H3B	0.8405	0.7649	0.2884	0.042*	
H3A	0.6524	0.6529	0.2941	0.042*	
C4	0.6288 (3)	0.8728 (3)	0.23202 (9)	0.0454 (5)	
H4B	0.6617	0.9895	0.2510	0.054*	
H4A	0.4946	0.8553	0.2358	0.054*	
C5	0.6903 (2)	0.8652 (2)	0.16126 (8)	0.0314 (4)	
H5A	0.5790	0.8736	0.1339	0.038*	
C6	0.8300 (3)	1.0102 (2)	0.14059 (8)	0.0331 (4)	
H6A	0.9284	1.0227	0.1731	0.040*	
C7	0.9168 (3)	0.9620 (2)	0.07610 (9)	0.0359 (4)	
H7A	0.8175	0.9664	0.0437	0.043*	
C8	1.0011 (2)	0.7695 (3)	0.07524 (8)	0.0341 (4)	
H8A	1.0387	0.7430	0.0310	0.041*	
C9	0.8502 (3)	0.6316 (2)	0.09368 (8)	0.0339 (4)	
H9B	0.7529	0.6298	0.0612	0.041*	
H9A	0.9043	0.5102	0.0963	0.041*	
C10	1.1765 (3)	0.7517 (3)	0.11756 (10)	0.0399 (4)	
H10B	1.2577	0.8557	0.1098	0.048*	
H10A	1.1390	0.7552	0.1623	0.048*	
C11	1.2854 (3)	0.5778 (3)	0.10545 (10)	0.0514 (5)	
H11C	1.3930	0.5743	0.1331	0.062*	
H11B	1.3255	0.5745	0.0615	0.062*	
H11A	1.2070	0.4741	0.1141	0.062*	
N1	0.7703 (2)	0.68207 (18)	0.15522 (7)	0.0292 (3)	
O21	0.8643 (2)	0.44131 (16)	0.21682 (6)	0.0420 (3)	
O22	0.7283 (2)	1.17774 (17)	0.13508 (8)	0.0473 (4)	
H22A	0.776 (4)	1.257 (4)	0.1571 (12)	0.057*	
O23	1.0557 (2)	1.0928 (2)	0.05882 (8)	0.0565 (4)	
H23A	1.015 (4)	1.177 (4)	0.0304 (13)	0.068*	
O24	0.9507 (2)	1.3468 (3)	−0.03262 (8)	0.0559 (4)	
H24A	1.013 (4)	1.312 (4)	−0.0684 (13)	0.067*	
H24B	0.851 (4)	1.369 (4)	−0.0451 (13)	0.067*	

### Atomic displacement parameters (Å^2^)

**Table:**

	*U*^11^	*U*^22^	*U*^33^	*U*^12^	*U*^13^	*U*^23^
C2	0.0274 (8)	0.0256 (8)	0.0373 (9)	−0.0042 (7)	−0.0022 (7)	−0.0007 (7)
C3	0.0365 (9)	0.0329 (8)	0.0346 (9)	−0.0024 (8)	0.0014 (7)	−0.0006 (7)
C4	0.0523 (11)	0.0395 (10)	0.0444 (11)	0.0134 (10)	0.0139 (9)	0.0010 (9)
C5	0.0312 (8)	0.0270 (8)	0.0359 (9)	0.0044 (7)	−0.0026 (7)	−0.0004 (7)
C6	0.0355 (9)	0.0251 (8)	0.0387 (9)	−0.0008 (8)	−0.0106 (7)	0.0016 (7)
C7	0.0325 (9)	0.0376 (9)	0.0375 (9)	−0.0057 (8)	−0.0061 (8)	0.0108 (8)
C8	0.0354 (9)	0.0404 (9)	0.0265 (8)	−0.0020 (8)	0.0011 (7)	0.0010 (7)
C9	0.0378 (9)	0.0327 (9)	0.0312 (9)	−0.0021 (8)	0.0004 (7)	−0.0064 (7)
C10	0.0323 (9)	0.0396 (10)	0.0478 (11)	−0.0019 (9)	−0.0005 (8)	0.0035 (9)
C11	0.0504 (12)	0.0576 (13)	0.0462 (12)	0.0150 (11)	−0.0002 (10)	0.0036 (10)
N1	0.0316 (7)	0.0230 (6)	0.0330 (7)	0.0003 (6)	0.0019 (6)	−0.0007 (6)
O21	0.0514 (8)	0.0282 (6)	0.0465 (7)	0.0091 (6)	−0.0047 (7)	0.0021 (6)
O22	0.0560 (9)	0.0234 (6)	0.0624 (9)	0.0034 (7)	−0.0181 (7)	−0.0010 (6)
O23	0.0418 (8)	0.0553 (9)	0.0726 (10)	−0.0098 (8)	−0.0016 (7)	0.0328 (8)
O24	0.0482 (9)	0.0650 (10)	0.0546 (9)	0.0009 (9)	0.0021 (7)	0.0157 (8)

### Geometric parameters (Å, °)

**Table:**

C2—O21	1.228 (2)	C7—H7A	0.9800
C2—N1	1.339 (2)	C8—C9	1.525 (2)
C2—C3	1.507 (2)	C8—C10	1.538 (3)
C3—C4	1.518 (3)	C8—H8A	0.9800
C3—H3B	0.9700	C9—N1	1.452 (2)
C3—H3A	0.9700	C9—H9B	0.9700
C4—C5	1.540 (2)	C9—H9A	0.9700
C4—H4B	0.9700	C10—C11	1.513 (3)
C4—H4A	0.9700	C10—H10B	0.9700
C5—N1	1.462 (2)	C10—H10A	0.9700
C5—C6	1.519 (2)	C11—H11C	0.9600
C5—H5A	0.9800	C11—H11B	0.9600
C6—O22	1.429 (2)	C11—H11A	0.9600
C6—C7	1.522 (3)	O22—H22A	0.82 (3)
C6—H6A	0.9800	O23—H23A	0.90 (3)
C7—O23	1.425 (2)	O24—H24A	0.91 (3)
C7—C8	1.532 (3)	O24—H24B	0.78 (3)
			
O21—C2—N1	125.27 (16)	C8—C7—H7A	107.9
O21—C2—C3	126.23 (16)	C9—C8—C7	109.13 (14)
N1—C2—C3	108.50 (14)	C9—C8—C10	112.01 (14)
C2—C3—C4	105.09 (15)	C7—C8—C10	113.01 (15)
C2—C3—H3B	110.7	C9—C8—H8A	107.5
C4—C3—H3B	110.7	C7—C8—H8A	107.5
C2—C3—H3A	110.7	C10—C8—H8A	107.5
C4—C3—H3A	110.7	N1—C9—C8	109.40 (14)
H3B—C3—H3A	108.8	N1—C9—H9B	109.8
C3—C4—C5	105.20 (14)	C8—C9—H9B	109.8
C3—C4—H4B	110.7	N1—C9—H9A	109.8
C5—C4—H4B	110.7	C8—C9—H9A	109.8
C3—C4—H4A	110.7	H9B—C9—H9A	108.2
C5—C4—H4A	110.7	C11—C10—C8	113.21 (17)
H4B—C4—H4A	108.8	C11—C10—H10B	108.9
N1—C5—C6	111.06 (13)	C8—C10—H10B	108.9
N1—C5—C4	103.10 (13)	C11—C10—H10A	108.9
C6—C5—C4	115.70 (15)	C8—C10—H10A	108.9
N1—C5—H5A	108.9	H10B—C10—H10A	107.7
C6—C5—H5A	108.9	C10—C11—H11C	109.5
C4—C5—H5A	108.9	C10—C11—H11B	109.5
O22—C6—C5	106.74 (14)	H11C—C11—H11B	109.5
O22—C6—C7	109.57 (15)	C10—C11—H11A	109.5
C5—C6—C7	110.85 (14)	H11C—C11—H11A	109.5
O22—C6—H6A	109.9	H11B—C11—H11A	109.5
C5—C6—H6A	109.9	C2—N1—C9	126.13 (14)
C7—C6—H6A	109.9	C2—N1—C5	114.67 (13)
O23—C7—C6	110.54 (16)	C9—N1—C5	117.56 (13)
O23—C7—C8	109.97 (14)	C6—O22—H22A	110.7 (18)
C6—C7—C8	112.57 (14)	C7—O23—H23A	113.7 (16)
O23—C7—H7A	107.9	H24A—O24—H24B	104 (3)
C6—C7—H7A	107.9		
			
O21—C2—C3—C4	−169.29 (17)	C6—C7—C8—C10	−68.92 (19)
N1—C2—C3—C4	11.57 (19)	C7—C8—C9—N1	−55.28 (18)
C2—C3—C4—C5	−17.83 (19)	C10—C8—C9—N1	70.64 (19)
C3—C4—C5—N1	17.48 (19)	C9—C8—C10—C11	69.5 (2)
C3—C4—C5—C6	−103.96 (16)	C7—C8—C10—C11	−166.77 (16)
N1—C5—C6—O22	167.96 (14)	O21—C2—N1—C9	−14.3 (3)
C4—C5—C6—O22	−74.98 (18)	C3—C2—N1—C9	164.90 (15)
N1—C5—C6—C7	48.69 (18)	O21—C2—N1—C5	−179.17 (16)
C4—C5—C6—C7	165.76 (15)	C3—C2—N1—C5	−0.02 (19)
O22—C6—C7—O23	65.90 (18)	C8—C9—N1—C2	−108.10 (19)
C5—C6—C7—O23	−176.56 (14)	C8—C9—N1—C5	56.43 (19)
O22—C6—C7—C8	−170.69 (13)	C6—C5—N1—C2	113.25 (16)
C5—C6—C7—C8	−53.14 (19)	C4—C5—N1—C2	−11.28 (19)
O23—C7—C8—C9	−179.85 (15)	C6—C5—N1—C9	−53.04 (19)
C6—C7—C8—C9	56.42 (18)	C4—C5—N1—C9	−177.56 (15)
O23—C7—C8—C10	54.81 (19)		

### Hydrogen-bond geometry (Å, °)

**Table:**

*D*—H···*A*	*D*—H	H···*A*	*D*···*A*	*D*—H···*A*
O22—H22A···O21^i^	0.82 (3)	1.94 (3)	2.7505 (19)	173 (3)
O24—H24A···O22^ii^	0.91 (3)	2.07 (3)	2.919 (2)	155 (2)
O24—H24B···O23^iii^	0.78 (3)	2.14 (3)	2.907 (2)	167 (3)

Symmetry codes: (i) *x*, *y*+1, *z*; (ii) *x*+1/2, −*y*+5/2, −*z*; (iii) *x*−1/2, −*y*+5/2, −*z*.
